# Quantitative proteomics and bioinformatic analysis provide new insight into the dynamic response of porcine intestine to *Salmonella* Typhimurium

**DOI:** 10.3389/fcimb.2015.00064

**Published:** 2015-09-03

**Authors:** Melania Collado-Romero, Carmen Aguilar, Cristina Arce, Concepción Lucena, Marius C. Codrea, Luis Morera, Emoke Bendixen, Ángela Moreno, Juan J. Garrido

**Affiliations:** ^1^Grupo de Genómica y Mejora Animal, Departamento de Genética, Facultad de Veterinaria, Universidad de CórdobaCórdoba, Spain; ^2^Departamento de Producción Animal, Facultad de Veterinaria, Universidad de CórdobaCórdoba, Spain; ^3^Department of Molecular Biology and Genetics, Faculty of Science and Technology, Aarhus UniversityAarhus, Denmark; ^4^Instituto de Agricultura Sostenible, Consejo Superior de Investigaciones CientíficasCórdoba, Spain

**Keywords:** iTRAQ, *Salmonella* Typhimurium, experimentally infected pigs, intestinal response, ileum, colon

## Abstract

The enteropathogen *Salmonella* Typhimurium (*S.* Typhimurium) is the most commonly non-typhoideal serotype isolated in pig worldwide. Currently, one of the main sources of human infection is by consumption of pork meat. Therefore, prevention and control of salmonellosis in pigs is crucial for minimizing risks to public health. The aim of the present study was to use isobaric tags for relative and absolute quantification (iTRAQ) to explore differences in the response to *Salmonella* in two segment of the porcine gut (ileum and colon) along a time course of 1, 2, and 6 days post infection (dpi) with *S.* Typhimurium. A total of 298 proteins were identified in the infected ileum samples of which, 112 displayed significant expression differences due to *Salmonella* infection. In colon, 184 proteins were detected in the infected samples of which 46 resulted differentially expressed with respect to the controls. The higher number of changes in protein expression was quantified in ileum at 2 dpi. Further biological interpretation of proteomics data using bioinformatics tools demonstrated that the expression changes in colon were found in proteins involved in cell death and survival, tissue morphology or molecular transport at the early stages and tissue regeneration at 6 dpi. In ileum, however, changes in protein expression were mainly related to immunological and infection diseases, inflammatory response or connective tissue disorders at 1 and 2 dpi. iTRAQ has proved to be a proteomic robust approach allowing us to identify ileum as the earliest response focus upon *S.* Typhimurium in the porcine gut. In addition, new functions involved in the response to bacteria such as eIF2 signaling, free radical scavengers or antimicrobial peptides (AMP) expression have been identified. Finally, the impairment at of the enterohepatic circulation of bile acids and lipid metabolism by means the under regulation of FABP6 protein and FXR/RXR and LXR/RXR signaling pathway in ileum has been established for the first time in pigs. Taken together, our results provide a better understanding of the porcine response to *Salmonella* infection and the molecular mechanisms underlying *Salmonella*-host interactions.

## Introduction

*Salmonella enterica* serovar Typhimurium is one of the most common causes of bacterial gastroenteritis in humans and many species of food producing farm animals (Boyen et al., 2008). In pigs, *S.* Typhimurium causes enterocolitis and frequently exits in a subclinical carrier state, in which infected animal will intermittently or continuously shed bacteria organisms in their feces for very prolonged periods of time, making elimination of the infection difficult (Gopinath et al., 2012). In humans, *S.* Typhimurium is the second serovar most frequently reported in the EU and infection by this pathogen is mostly associated with the consumption of contaminated pork (Foley and Lynne, 2008).

Although the gastrointestinal tract is considered to be their biological niche, *Salmonella* preferentially colonize ileum, caecun and colon (Darwin and Miller, 1999; Boyen et al., 2008). Pathogen interaction with the intestinal epithelium and the underlying resident immune cells will initiate a host response to face up the infection. At this point the early action of the innate immune response and the subsequent engagement of the acquired response will be crucial to the control of the infection (Ravindran and McSorley, 2005; Grassl and Finlay, 2008). Nevertheless, *Salmonella* is a very successful enteric pathogen that has developed multiple virulence strategies to evade and modulate most of the immune defenses employed by the host (Ruby et al., 2012). Investigating *Salmonella* pathogenesis in persistently infected mice has significantly contributed to our understanding of how bacteria respond to the host defense mechanisms. Thus, mice models of *S.* Typhimurium infection have revealed that *Salmonella* can replicate within the phagocytes and remains partially hidden within its intracellular niche (Buckner and Finlay, 2011). Also, recent reports have shown that *Salmonella* use effector proteins to manipulate the host inflammatory response in order to persist in mammalian cells (McSorley, 2014). Unlike in mice, where *S.* Typhimurium causes a systemic infection, bacteria usually cause both in humans and pigs a self-limiting gastrointestinal disease. Consequently, pig is considered as one of the most compatible models for *in vivo* studies of human intestinal infections (Kararli, 1995; Wernersson et al., 2005). The pig model not only will contribute to the understanding to *Salmonella* pathogenesis in intestine of these animals of great importance in agronomy but also might shed light of some aspects of *Salmonella* infections in humans.

The transcriptional response of the porcine intestine to *Salmonella* has been reported in several studies (Meurens et al., 2009; Collado-Romero et al., 2010; Hulst et al., 2013) while information at the proteome level is still limited (Soler et al., 2014). Recently, a couple of studies have been conducted by our group which have addressed *in vivo* porcine response to *S.* Typhimurium infection using gel-based approaches (Collado-Romero et al., 2012; Arce et al., 2014). The obtained results in both experimental and naturally infected animals suggest that host response to *Salmonella* infection involves processes related to inflammation, remodeling of the cytoskeleton and metabolism and that this response seems to be modulated in an active way by the pathogen. To reach greater knowledge about the mechanism developed by *Salmonella* to evade the host response as well as to analyze and compare the response to infection in different anatomical portions of the porcine gut, in the present study, the expression levels of differentially expressed proteins in ileum and colon from pigs infected with *S.* Typhimurium were analyzed using isobaric tags for relative and absolute quantitation (iTRAQ). The biological significance of identified differentially expressed proteins were further evaluated using bioinformatic tools in order to provide a full picture of the events triggered by *S.* Typhimurium in pigs.

## Materials and methods

### Experimental infection and tissue collection

Details of the experimental infection have been already reported (Collado-Romero et al., 2010). Briefly, 12 *Salmonella*-free piglets of 4 weeks of age were orally infected with 10^8^ cfu of *S. enterica* serovar Typhimurium phagetype DT104 whereas the control group (four animals) received sterile medium. The four non-infected control pigs were necropsied prior to experimental infection, and subsequently, four randomly chosen infected pigs were necropsied at 1 day post infection (dpi), 2 dpi, and 6 dpi. Sections from ileum and colon were collected and immediately frozen in liquid nitrogen for subsequent mucosa isolation and protein extraction. Piglets were housed in the experimental isolation facilities of the University of León (Spain). Animal care and procedures were in accordance with the guidelines of the Good Experimental Practices, under the supervision of the Ethical and Animal Welfare Committee of the University of León.

### Histological analysis

Formalin-fixed tissues were embedded in paraffin wax following standard procedures. Sections of 5 μm were placed on slides coated with Poly-l-Lysine (Sigma–Aldrich) and kept at 55°C for 45 min. Slides were dewaxed in xylene and rehydrated through graded alcohols to distilled water and stained with hematoxylin and eosin (H&E). For immunohistochemistry, slides were then subjected to heat-mediated antigen retrieval in 0.01 M citric acid, incubated with a polyclonal antibody developed in our laboratory against *Salmonella* and stained as described before (Martins et al., 2012).

### Mucosa isolation and protein extraction

Intestinal sections of around 2 cm were thawed onto an ice-cold plate and opened by means of a longitudinal cut. All process was carried out at 4°C. Luminal surface was thorough cleaned using sterile gauze and PBS to eliminate mucus and blotted dry onto dried gauze. Mucosa scrapings were obtained using a razor, weighted and homogenized in lysis buffer (10 mM Tris-HCl, pH 7.6; 1 mM EDTA, 0.25 M sucrose; 1:100 (vol/vol) Proteinase Inhibitor Cocktail P8340 (Sigma-Aldrich) using pre-chilled mortar and pestles. Lysis buffer was used in a proportion of 1 ml per 250 mg of mucosa. Homogenized samples were incubated for 20 min in an orbital shaker and finally centrifuged at 10,000 × g for 30 min at 4°C. The supernatant was stored at −80°C until use. Protein concentrations were determined using the Pierce BCA Protein Assay Kit (Cultek SL, Madrid, Spain) using BSA as standard according to manufacturer's instructions.

### iTRAQ assay design and peptide labeling

iTRAQ is a protein identification and quantitation technique that utilizes different isobaric amine specific tags to label the primary amines of peptides from different samples. The samples (control and different conditions to study) are digested and peptides from each are labeled with a different tag. These labeled peptides allow determining relative protein levels, with reference to a control state. In this study four 4-plex iTRAQ analyses were performed per intestinal section and samples were labeled with tags 114, 115, 116, and 117. Firstly, a common reference sample was generated by pooling proteins from the four controls-non inoculated animals. Then, four aliquots of this pool were used for independent labeling with mass 114 and randomly assigned to 1, 2, 3, and 4 replicates. Infected samples after 1, 2, or 6 dpi were labeled with masses 115, 116, and 117, respectively and randomly assigned to replicates 1, 2, 3, and 4. Proteins digestion and peptide labeling with iTRAQ reagents was carried out according to the AB Sciex protocol for a 4-plex procedure (AB Sciex, Foster City, CA, USA) as previously described (Pedersen et al., 2010). Briefly, 100 μg of protein from each sample was resuspended in 20 μL of digestion buffer (0.5 M triethyl-ammonium-bicarbonate, 0.1% sodium dodecyl sulfate) and cystein residues was reduced with Tris (2-carboxyethyl) phosphine hydrochloride and capped with methylmethanethiosulfate (MMTS), before overnight digestion with trypsin (Promega). Isobaric tagging iTRAQ reagent (1 U in ethanol) was added directly to the protein digests and incubated at room temperature for 1 h. Labeled control and infected samples were combined in 1:1:1:1 ratios into 4-plexed samples, each containing a common control sample and a sample for each of the three time points of infection. Ileum and colon samples were independently analyzed. The eight 4-plexed samples generated in this study (four for ileum and four for colon) were aliquoted into 55 μl (corresponding to about 55 ng), vacuum-dried and stored at −80°C until their afterward fractionation and analysis.

### Sample fractionation and mass spectrometry analysis (LC-MS/MS)

Each iTRAQ 4-plexed sample was fractionated using a strong cation exchange (SCX) column on an Agilent 1100 Series capillary HPLC equipped with a Zorbax Bio-SCX Series II, 0.8 × 50 mm column (Agilent Technologies, Palo Alto, CA). Eluted peptides were pooled into 11 fractions and further analyzed on Qstar Pulsar™ (Applied Biosystems-MDS Sciex) interfaced with an Agilent 1100 Series nanoflow HPLC system (Agilent Technologies, Palo Alto, CA) prior to mass spectrometric identification. The systems were equipped with an isocratic pump working at 20 μL/min (0.1% FA and 3% acetonitrile in water) for fast sample loading onto an enrichment-column (Zorbax 300SB C18, 0.3 × 5 mm, 5 μm particle, Agilent Technologies). This ensured sample de-salting and concentration. The HPLC system was directly connected with the mass spectrometer and the eluting peptides were sprayed through a nanospray needle (Fused Silica Emitters, OD 360μm, ID 75μm, Proxeon Biosystems, Odense, Denmark) into the Q-star Elite mass spectrometer (Applied Biosystems).

### Protein identification and data analysis

Relative abundance quantification and peptide and protein identification were performed using the ProteinPilotTM Software 1.0 (Applied Biosystems) using the ProGroup and Paragon algorithms for protein grouping and confidence scoring. Each MS/MS spectrum was searched against the mammalian KBMS5.0.20050302 protein database from Celera Discovery Systems (Applied Biosystems). The search parameters allowed for cysteine modification by methyl methanethiosulfonate and biological modifications programmed in the algorithm (i.e., amidations, phosphorylations, and semitryptic fragments). The protein confidence threshold cutoff was set to 1.3 (unused), with at least more than two peptides above the 95% confidence level and the tolerance settings for peptide identification were 0.15 Da for MS and 0.1 Da for MS/MS. Quantitation of proteins was relative to the common pooled sample present in each iTRAQ-MS experiment. Quantitative data were obtained with a minimum of two peptides per protein and using unique peptides. Identified proteins from the four replicates were merged. Proteins were grouped using both Panther ID and SwissProt accession number to ensure correct grouping of identical proteins. In order to give more robustness to our results only proteins that were identified and quantified in the four replicates were included in further data analysis. To compare the differential expression of one protein in different groups, we conducted a statistical analysis of quantitative data using the standard one-way ANOVA procedure in R (R Development Core Team, http://www.R-project.org) of the four replicate, establishing significant variance from ratio = 1 at a *p*-value < 0.05 and identifying the differentially expressed proteins among those whose ratios differed significantly from 1. The use of a common reference control (114) in the four multiplex-labeling allowed us to perform a statistical analysis across all the four iTRAQ experiments within a section. In order to compare identified proteins among intestinal sections, i.e., ileum and colon, we used first accessions as IDs and then recheck using the corresponding gene names.

### Western blot analysis

Proteins extracts (20 mg) from each of the two conditions compared (control and infected samples) were loaded on 12% SDS-PAGE and transferred onto a PVDF membrane (Millipore, Bedford, MA, USA). Membrane were blocked for 1 h with 5% dried milk in TBS buffer with 0.05% Tween-20 (TBS-T) and incubated overnight at 4°C with rabbit polyclonal antibody anti-14-3-3 β (Santa Cruz Biotechnologies, Santa Cruz, CA, USA) or anti-FKBP4 (Abcam, Cambridge, UK) at 1:1000 v/v dilution in 5% skimmed milk in TBS-T. Secondary peroxidase-linked anti-rabbit antibody (1:10000 dilution) was used to generate immunocomplexes, that were visualized with Immobilon Western chemiluminescent HRP substrate (Millipore). Triplicate blots were carried out for both proteins. Optical densities of the immunoreactive bands were measured using ImageJ 1.43u software. Statistical differences between groups were evaluated using a two-tailed *t*-test. To confirm equal sample loading, all membranes were reblotted with anti-GAPDH monoclonal antibody (GenScript, Picastaway, NJ, USA).

### Functional analysis of the proteins

Functional analysis of proteomics data were carried out using GeneCodis (http://genecodis.cnb.csic.es) and Ingenuity Pathway Analysis (IPA, Ingenuity Systems, www.ingenuity.com) as previously described (Ramírez-Boo et al., 2011). Previously, the accession numbers from NCBI, ENSEMBL, or Uni-Prot database were manually searched and associated to corresponding gene names. IPA can generate the functional analysis and networks that are most significant to the data set. This software compares proteins in the input group and displays a rank-ordered list of pathways whose activities are most likely affected (Esteso et al., 2008; Jiménez-Marín et al., 2009). Fisher's exact test was used to calculate a *p*-value determining the probability that each biological function assigned to the data set is due to change alone. These *p*-values are calculated based on the number of proteins that participate in a given pathway relative to the total number of occurrences of these proteins in all pathway annotations stored in the Ingenuity Pathways Knowledge Base. After loading data, not recognized or unmapped gene names were rechecked and synonyms gene names or IDs were used when available.

## Results

### *In Vivo S.* Typhimurium infection and histopathological analysis

**Figure 1:**
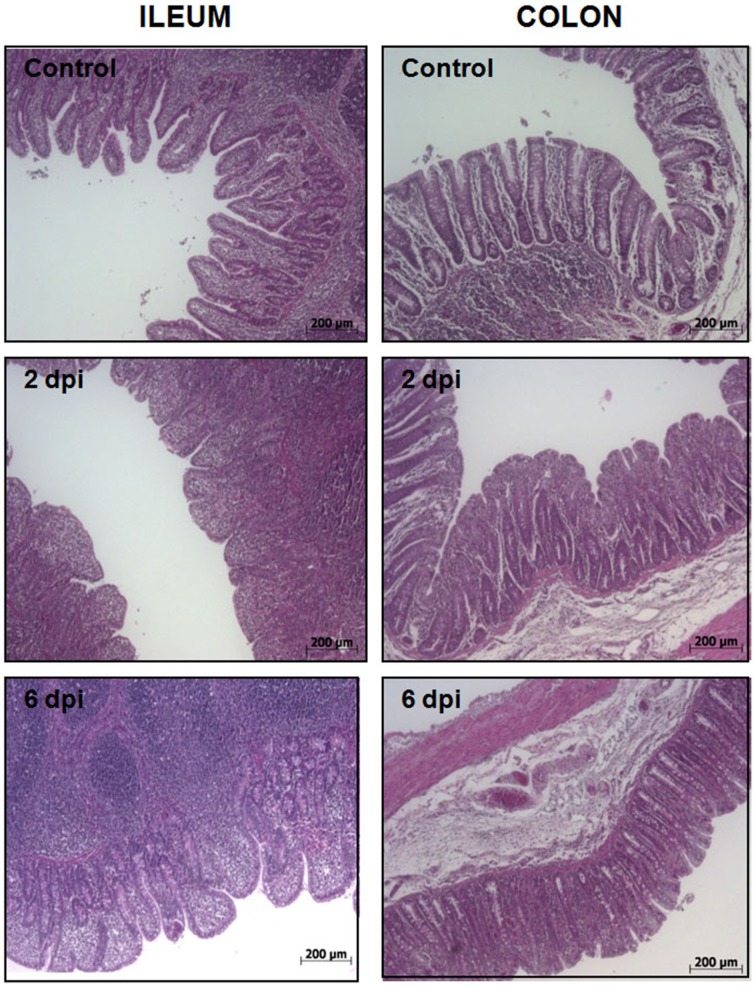
**Histological analysis of porcine intestinal mucosa upon *S.* Typhimurium infection**. H&E staining of ileum (left) and colon mucosa (right) from control, 2 dpi, and 6 dpi animals as indicated. Original magnification: 50X.

**Figure 2:**
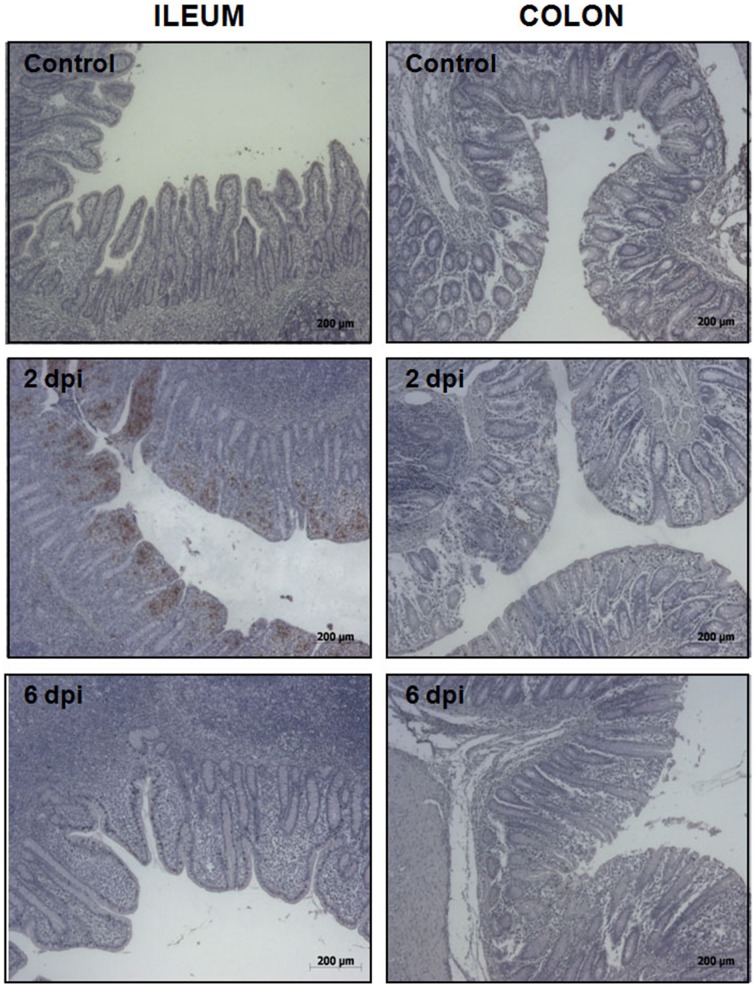
**Immunohistochemical detection of *S.* Typhimurium in ileum (left) and colon mucosa (right) from control, 2 dpi, and 6 dpi animals as indicated**. Original magnification: 50X.

To uncover the host response to *Salmonella* infection in different segments of the porcine gut, an *in vivo* model consisting of piglets orally challenged with *S.* Typhimurium was established (Collado-Romero et al., 2010). All the infected animals were fecal-culture positive for *Salmonella* and developed similar clinical signs of gastrointestinal disease, including increased rectal temperature, diarrhea and lethargy. The interaction of *Salmonella* with ileum and colon mucosa was analyzed by H&E staining and immunohistochemical detection using a specific antibody. H&E-stained tissue sections revealed extensive pathological changes in the intestinal mucosa, mainly at the early stage of infection (Figure 1). Thus, while several inflammatory features were observed at 2 dpi, including villi degradation in ileum and crypt destruction in ileum and colon, a substantial recovery of the mucosa structure was observed in both sections at 6 dpi. In accordance with this, immunostaining data showed a widespread colonization of *S.* Typhimurium in ileum at 2 dpi, mostly being restricted to the apical area of the mucosa (Figure 2). In contrast, *Salmonella* was poorly detected in ileum mucosa at 6 dpi as well as in colon, where bacterial colonization was substantially reduced. These results agreed with clinical signs such as fever, lethargy, weight loss and diarrhea which peaked in infected pigs at 2 dpi. On the contrary, clinical improvement of disease was noticed along the time of infection, with animals showing feces free from bacteria and normal body temperature at 6 dpi.

### Differential expressed proteins after *S.* Typhimurium infection

**Figure 3:**
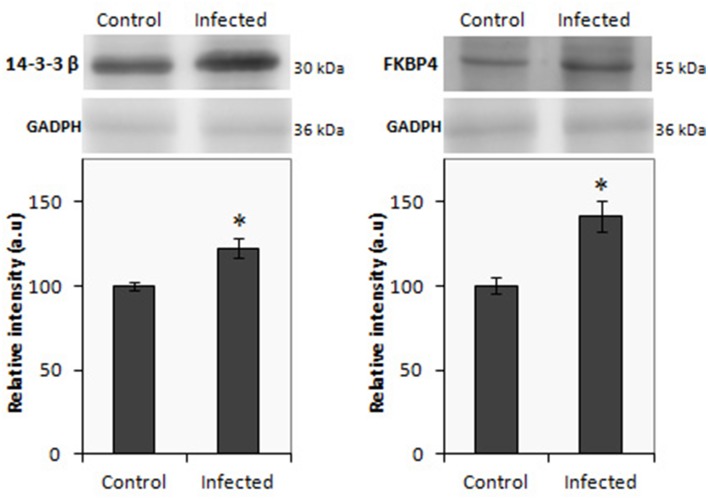
**Western blot of 14.3.3 β (left) and FBPK4 (right) using protein lysate from ileal and colon mucosa, repectively from infected and uninfected pigs**. The relative molecular mass of the proteins is given in kDa. Results are expressed in arbitrary units; ^*^p < 0.05.

In the present study, iTRAQ technology in combination with LC-MS/MS was applied to investigate differentially expressed proteins in the gut of *Salmonella* infected pigs. In ileum from infected animals, a total of 298 proteins were identified (Supplementary Table 1), of which 112 proteins displayed significant differences in expression levels at least in one time point. As shown in Table 1, 55 proteins were up-regulated and 56 down-regulated. A total of 184 proteins were detected in infected colon samples (Supplementary Table 2) of which 46 resulted differentially expressed. Among these proteins, 21 were up regulated proteins and 25 proteins resulted down regulated after the *Salmonella* infection. The higher number of changes in protein expression was measured in ileum, at 2 dpi and only 19 proteins were differentially expressed in both intestinal sections in at least one of the analyzed time points (Supplementary Table 3). Two proteins 14.3.3 ß and FKBP4 were selected to validate the alteration trend by western blot using ileum and colon samples from infected animals, respectively. Consistent with the proteomics results, as shown in Figure 3, western blot analysis confirmed that the expression of both proteins significantly increased in the infected tissues when compared with control samples.

**Table 1:** **Analysis of the canonical pathways corresponding to the data set obtained**.

**1 dpi**	***p*-value**	**2 dpi**	***p*-value**	**6 dpi**	***p*-value**
**CANONICAL PATHWAYS (ILEUM)**					
EIF2 signaling	1,72E-05	EIF2 signaling	1,75E-15	EIF2 signaling	2,69E-04
Regulation of actin-based motility by Rho	4,14E-04	Regulation of actin-based motility by Rho	3,18E-05	TCA cycle	7,13E-04
RhoA signaling	1,17E-03	Remodeling of epithelial adherens junctions	7,34E-04	Gluconeogenesis	7,77E-04
Epithelial adherens junction signaling	1,15E-03	eNOS signaling	5.92E-04	Aldosterone signaling in epithelial cells	2,24E-03
Calcium signaling	1,12E-03	Calcium signaling	2,05E-04	Actin cytoskeleton signaling	2,19E-03
FXR/RXR activation	1,01E-03	LXR/RXR activation	8.92E-03	eNOS signaling	1,46E-02
**CANONICAL PATHWAYS (COLON)**					
Aldosterone signaling in epithelial cells	1,83E-03	Aldosterone signaling in epithelial cells	1,79E-06	B cell development	9,14E-05
Protein ubiquitination pathway	7,81E-03	Protein ubiquitination pathway	2,22E-05	PI3K signaling in B lymphocytes	5,22E-02
14-3-3-mediated signaling	8,79E-02	PI3K/AKT signaling	2,28E-05	eNOS signaling	4,78E-02
eNOS signaling	7,25E-02	eNOS signaling	2,92E-05	Aldosterone signaling in epithelial cells	3,90E-02

The analysis is derived from the Ingenuity Pathways Analysis.

### Biological interpretation of the significantly altered proteins

**Figure 4:**
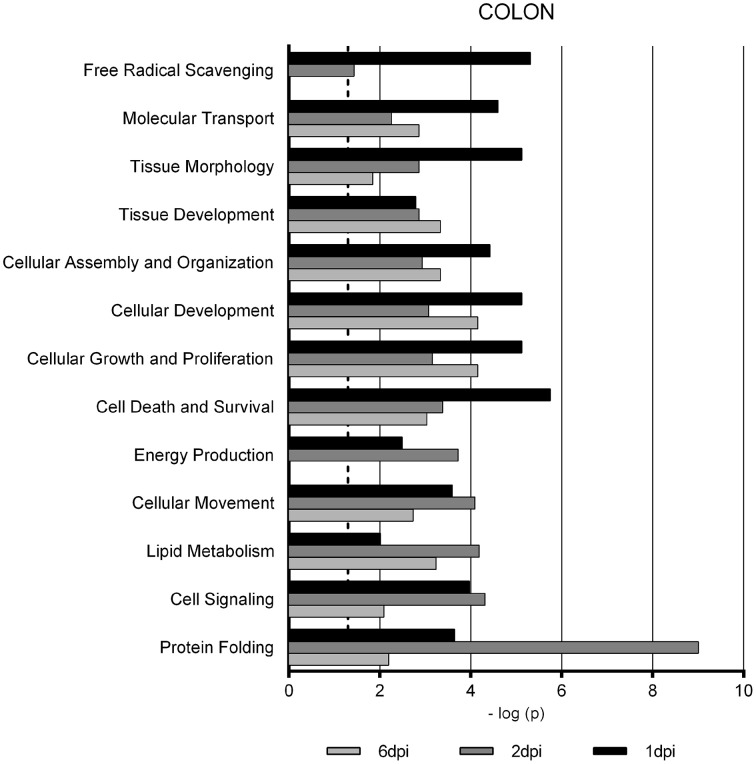
**Enriched functions associated to the response of porcine colon to *S.* Typhimurium infection**. The analysis is derived from the Ingenuity Pathways Analysis.

**Figure 5:**
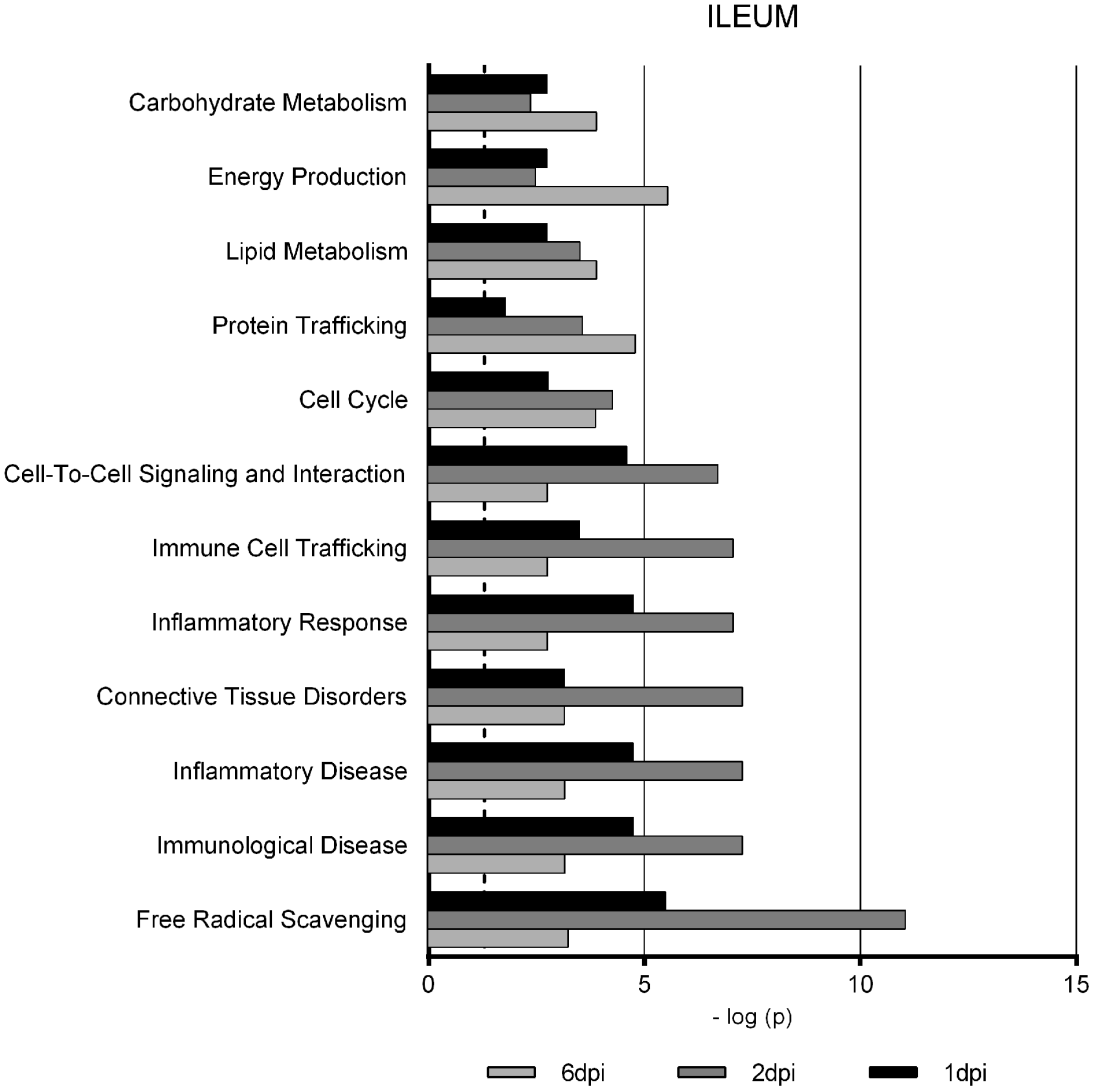
**Enriched functions associated to the response of porcine ileum to *S.* Typhimurium infection**. The analysis is derived from the Ingenuity Pathways Analysis.

Gene Ontology studies using Ingenuity Pathway Analysis software classified the significantly altered proteins as per their biological functions as well as ranked them as per their involvement in certain canonical pathways. In colon at 1 dpi we found changes in the expression of proteins implicated in cell death and survival, tissue morphology, molecular transport or free radical scavenging among others while at 2 dpi the changes in functions such as protein folding, cell signaling, lipid, and nucleic acid metabolism or energy production were predominant (Figure 4). At 6 dpi, however, functions required to recovery tissue structure such as cellular development, growth and proliferation or tissue development were significantly altered. In ileum, the infection with *Salmonella* caused changes in different functions, being immunological and infection diseases, inflammatory response or free radical scavenging the most significantly altered functions at 1 dpi (Figure 5). In addition to these functions, at 2 dpi we also found changes in the expression of proteins involved in cellular movement, connective tissue disorders, and immune cell trafficking. It was noted, however, that function related with the bacterial immune response has less significance while gaining prominence basic functions of the cell such as energy production and metabolism, protein synthesis, and trafficking or cell cycle. Pathway analysis based in the IPA program was also performed. Table 1 shows the most significant canonical pathways enriched by proteins that were significantly differentially expressed in ileum and colon of pigs following oral *Salmonella* infection. Interestingly, EIF2 signaling, a pathway directly linked with infectious and inflammatory processes resulted significantly altered along the time course of infection. FXR/RXR and LXR/RXR activation, both pathways involved in lipid metabolism resulted impaired by the bacteria at 1 and 2 dpi, respectively and Rho signaling pathway involved in the actin regulation and remodeling of cellular junction were altered also at the early stages of infection (1 and 2 dpi). In colon, the association of the proteins deregulated with canonical pathways highlighted two pathways altered along the time course of the infection: aldosterone signaling in epithelial cells and eNOS signaling while other pathways such as B cell development and PI3K/AKT signaling changed only in some stage of the infection.

## Discussion

Despite the advances in understanding the mechanisms involved in host–pathogen interactions as well as the host cell responses to *S.* Typhimurium, the global proteome changes that occur in infected pigs at intestinal level have been poorly characterized. Researches in these issues are sustained by the need to develop vaccine and better diagnostic techniques. Although data from colonization studies indicate that *S.* Typhimurium preferentially colonize ileum and colon and less frequently jejunum (Boyen et al., 2008; Collado-Romero et al., 2010), the differential response to this enteric pathogen in distinct segments of the porcine intestine has not been entirely characterized. In Using in-gel based proteomic methods, in previous studies we have analyzed the early response to *S.* Typhimurium in the ileum of experimental infected pigs (Collado-Romero et al., 2012) and the changes in protein expression that occur in the intestinal mucosa of naturally infected pigs (Arce et al., 2014). Here, we analyze and compare the response to *S.* Typhimurium in two anatomical portions of the porcine gut (ileum and colon) using an iTRAQ based proteomic approach. The obtained results confirmed our previous evidences indicating that, in the swine response to salmonellosis are of great relevance and potential biological significance certain features such as the inflammatory response, Rho A signaling and cytoskeleton remodeling. In addition, iTRAQ has proved to be a more robust approach than other proteomic approaches, obtaining a greater number of differentially expressed proteins that have allowed us to identify ileum as the earliest response focus upon *S.* Typhimurium in the porcine gut and new functions involved in the response to bacteria in pigs.

Thus, the analysis with bioinformatic tools showed that free radical scavenging is the most significantly function altered in ileum at 1 and 2 dpi because protein such as galectin 3, apolipoprotein A-I, annexin 1, superoxide dismutase (SOD), or peroxiredoxin 1 has been differentially regulated after the infection. Free radicals, including reactive oxygen species (ROS), are known to play a vital role in salmonellosis (Bayim et al., 2012). While ROS are produced during the progress of salmonellosis, in association with an increase of oxidative stress, excessive amounts can cause deleterious effects in the intestinal tissue. Peroxiredoxin 1 and SOD attenuate the excess of free radical thus decreasing its concentration and activity and therefore play an important role in the protection of tissue damage. Intestinal injury and colitis is also caused by the activation of protein kinase C (PKC) mediated by reactive oxidant and this injury could be also ameliorated by SOD (Tepperman et al., 2000). In our study, SOD and peroxiredoxin 1 resulted down-regulated after *Salmonella* infection in ileum at 1 and 2 dpi, whereas PKC was found up-regulated. These changes could indicate that PKC activation caused by free radical and oxidative stress, along with the decrease in the expression of oxidant scavengers could have an important role in the extensive tissue damage caused by *Salmonella* in ileum at the early time of the infection. Accordingly, the lower tissue damage observed at 6 dpi seems to be associated with normal levels of PKC and SOD expression. This effect appears to be exclusive to ileum, since none of these proteins, functions or canonical pathways resulted altered in colon after *Salmonella* infection. On the other hand, activation of PKC has been implicated in cellular injury caused by high concentration of nitric oxide (NO) (Jun et al., 1997). NO is a potent host defense effector with antimicrobial activity but its overproduction cause host tissue damage and disturb normal gastrointestinal function (Bódi et al., 2014; Yao et al., 2014). NO is generated by nitric oxide synthase (NOS) in several immune cells such as macrophages or epithelial cells. NOS signaling is another canonical pathways altered by *Salmonella* infection at 2 and 6 dpi in colon and ileum. Proteins differentially expressed in this study, such as calmodulin or different heat shock proteins (HSP), are involved in this pathway. In addition, the macrophage migration inhibitory factor (MIF) was down regulated at 2 dpi in ileum. MIF is implicated in NO production, which is rapidly released after exposure to bacterial toxins. This protein protects the cells from inflammation-associated cell damage (Damico et al., 2008) and could be contribute to the cellular damage observed at 2 dpi.

Several studies have suggested that eIF2 signaling, together with PKR (double-stranded RNA-activated protein kinase) is activated during bacterial infection (Leung et al., 2014) constituting an important mechanism for adapting to stress condition (Pervin et al., 2008). They are involved in the innate immune response (Shrestha et al., 2012), protecting the host cell by blocking translation of pathogen mRNAs (Kim et al., 2011). EIF2 signaling was the top canonical pathway statistically enriched at 1, 2, and 6 dpi after *Salmonella* infection. Most of the proteins involved in this pathway, such as EEF1A (eukaryotic translation elongation factor 1 alpha 1) or different ribosomal proteins, resulted under-expressed at the early stages of the infection which could indicate that *Salmonella* is able to develop strategies to override the cellular response with the aim to survive into the host. This would agree with the successful *Salmonella* colonization found in ileum at 2 dpi, as well as the lack of inflammatory response previously observed by us in this intestinal segment after infection (Collado-Romero et al., 2010). EIF2 and PKR signaling has been also related with the role of HSP27 and HSP70 in mucosal inflammation (Hu et al., 2007) and both proteins have resulted altered in our study. In addition, it has been suggested that these pathways facilitate NF-kB activation under stress condition (Wek et al., 2006), participate in caspase-dependent apoptosis (Saelens et al., 2001) and macrophage death by pyroptosis (Lu et al., 2012). Both apoptosis and pyroptosis are processes that have been previously related with the porcine response to *Salmonella* (Martins et al., 2012), thus confirming the important role of eIF2 and PKR signaling in the infection process generated by *Salmonella* in swine.

Changes in the expression of different antimicrobial peptides (AMP) have been observed in ileum after *Salmonella* infection. AMP are important components of the innate immunity with antimicrobial activity interacting with the lipid matrix of bacterial membrane (Wessely-Szponder et al., 2010). AMP expression is regulated by NF-κB at transcriptional level (Linde et al., 2008). Therefore, the expression of AMP in ileum is in accordance with the NF-κB signaling activation previously observed after *Salmonella* infection (Collado-Romero et al., 2010). Here, S100A8 and S100A9, two members of the S100 calcium-binding protein family of proteins (Donato et al., 2013), resulted up-regulated at 2 dpi, stage in which we find a higher level of bacterial colonization in the mucosa as well as greater alteration in the morphology of the tissue. Calprotectin (a heterodimer of S100A8 and S100A9) expression inhibits *S.* Typhimurium (Nisapakultorn et al., 2001) and other pathogens such as *Listeria monocytogenes* or *Porphyromonas gingivalis* (Champaiboon et al., 2009). Our results are in agreement with previous studies showing that when the level of *Salmonella* increases in the intestine, calprotectin expression is induced in response (Zaia et al., 2009). Prophenin-1 is another AMP found differently expressed after *Salmonella* infection. This protein was first identified in porcine leukocytes and exhibits *in vivo* activity substantially against Gram negative bacteria (Harwig et al., 1995). The expression of prophenin-1 was the highest in our dataset and therefore the role that this protein might play in the porcine response to *Salmonella* infection deserves to be studied. In colon, none of these AMP has result differentially expressed after infection, suggesting that there is a differential pig antimicrobial response in both intestinal sections. Nevertheless, in colon we found changes in the expression of the histones H1 and H2B, which exhibit antimicrobial activity in addition to their established functions (Howell et al., 2003; Kawasaki and Iwamuro, 2008). These histones have been shown to have antimicrobial activity against different bacteria including S. Typhimurium (Rose et al., 1998), although its mode of action is different from that described for channel forming AMP such as S100 family of proteins or prophenin-1 (Aspedon and Groisman, 1996).

Differences between colon and ileum in response to *Salmonella* were also related to the changes in the aldosterone signaling pathway observed exclusively in colon as a consequence of the infection. Electrically tight epithelial monolayers, such as colon are considered classic aldosterone target tissues (Booth et al., 2002). Aldosterone is involved in the regulation of electrolyte and water balance through its effects on ion transport in epithelial cells. Phosphatidylinositol 3-kinases (PI3K) have been implicated in aldosterone signaling and we have found that PI3K/AKT signaling is also regulated by *Salmonella* infection (Wang et al., 2001). This fact is not surprising since electrolyte balance is altered in salmonellosis due to diarrheal episode associated with the disease.

Interestingly, iTRAQ results showed that different proteins and canonical pathways involved in lipid metabolism were also impaired after *Salmonella* infection. Thus, ileal lipid-binding protein (gastrotropin, FABP6) and apolipoprotein A-1 were observed to be down-regulated in the early stages of the infection. Also, FXR/RXR and LXR/RXR signaling pathways resulted also altered in response to bacteria at these stages. FABP6 is a member of the fatty acid binding protein (FABP) family and takes part in the enterohepatic circulation of the bile acids (Besnard et al., 2002). Bile acids are synthesized in the liver prior to be secreted into bile. After, they are secreted to the intestinal lumen and reabsorbed in the ileum to return to the liver closing a cycle of active transport. This process is regulated by activation of the farnesoid-X-receptor (FXR) and its interaction with retinoid-X-receptor (RXR) (Agellon et al., 2002) FABP6 gene expression is lost when mice are made deficient in FXR. So, down-regulation of these proteins and pathways in our experimental infection assay might be indicating that in pigs, *Salmonella* impaired enterohepatic circulation of bile acids and lipid metabolism for its own benefit, probably in order to form a niche for colonization in the intestine. In colon, none of these proteins or pathways has been altered, but FABP4, other protein belonging to the FABP family, has result overexpressed after the infection. FABP4 is a protein involved in lipid transport, and also related with the regulation of the inflammatory response (Makowski et al., 2005). The role of the FABP proteins in the response to *Salmonella* is still unknown. However, it is worth focusing on these proteins which function in the bacterial pathogenesis in order to better understand which function plays the lipid metabolism during the *Salmonella* infection.

In conclusion, applying iTRAQ to study the porcine intestinal response to *Salmonella* has allowed the identification and quantitation of 112 and 46 proteins in ileum and colon, respectively. These longer lists of proteins complement our previous gel-based proteomic study with a better proteome coverage allowing us to characterize the differences in response to bacteria in different section of the porcine intestine. This large-scale proteomic study provides a complete view of the major and important pathways which evolve during the infection process, thus providing a better understanding of the mechanisms of response developed by the host. In addition, there have also been identified a variety of physiological host processes targeted by *Salmonella* during the course of infection to facilitate to create a niche for their own survival in the porcine intestine.

## Author contributions

MC was responsible for the whole study, including lab work and iTRAQ data analysis, and contributed to writing of the manuscript. CrA and LM performed bioinformatics analysis. CrA performed the experimental infection and collected the tissue samples. CL participated in the histological analysis. MC and EB performed iTRAQ processing and statistical analysis. AM performed western blot analysis and participated in the writing of the manuscript. JG conceived and designed the project and participated in the interpretation and discussion of the results, as well as in the writing of the manuscript. All authors read and approved the final manuscript.

### Conflict of interest statement

The authors declare that the research was conducted in the absence of any commercial or financial relationships that could be construed as a potential conflict of interest.

## Supplementary material

Supplementary Table 1 **Proteins identified in ileum using iTRAQ analysis**.

Supplementary Table 2 **Proteins identified in colon using iTRAQ analysis**.

Supplementary Table 3 **Differentially regulated proteins in ileum and colon in response to Salmonella Typhimurium**. (↑) Proteins up-regulated after infection. (↓) Proteins down-regulated after infection (p<0.05).
